# Molecular species identification boosts bat diversity

**DOI:** 10.1186/1742-9994-4-4

**Published:** 2007-02-12

**Authors:** Frieder Mayer, Christian Dietz, Andreas Kiefer

**Affiliations:** 1 University of Erlangen; Department of Zoology; Staudtstrasse 5; D-91058 Erlangen; Germany; 2 University of Tübingen; Department of Animal Physiology; Auf der Morgenstelle 28; D-72076 Tübingen; Germany; 3 University of Mainz; Department of Zoology; Becherweg 13; D-55099 Mainz; Germany

## Abstract

The lack of obvious morphological differences between species impedes the identification of species in many groups of organisms. Meanwhile, DNA-based approaches are increasingly used to survey biological diversity. In this study we show that sequencing the mitochondrial protein-coding gene NADH dehydrogenase, subunit 1 (*nd1*) from 534 bats of the Western Palaearctic region corroborates the promise of DNA barcodes in two major respects. First, species described with classical taxonomic tools can be genetically identified with only a few exceptions. Second, substantial sequence divergence suggests an unexpected high number of undiscovered species.

## Findings

A total of at least 1.5 million species of organisms are described, but an estimated number of 10 to 100 million species may live on our planet [1,2]. This huge gap gave rise to search for new technologies to describe the biological diversity. Meanwhile, genetic screening approaches involving a single or a small number of genes – often referred to as DNA barcoding – were started in diverse taxonomic groups for two major aims: (i) to establish a standardized technique to identify species, and (ii) to detect new species in an efficient manner to bring us closer to the true number of species [3-6]. So far, DNA barcodes were primarily used for genetic species identification of taxonomically poorly studied taxa and geographic regions like the tropics [7-11]. Here we tested the applicability of DNA sequencing for species delineation and species identification in one of the best-known taxonomic groups in an intensively sampled geographic region.

**Figure 1:**
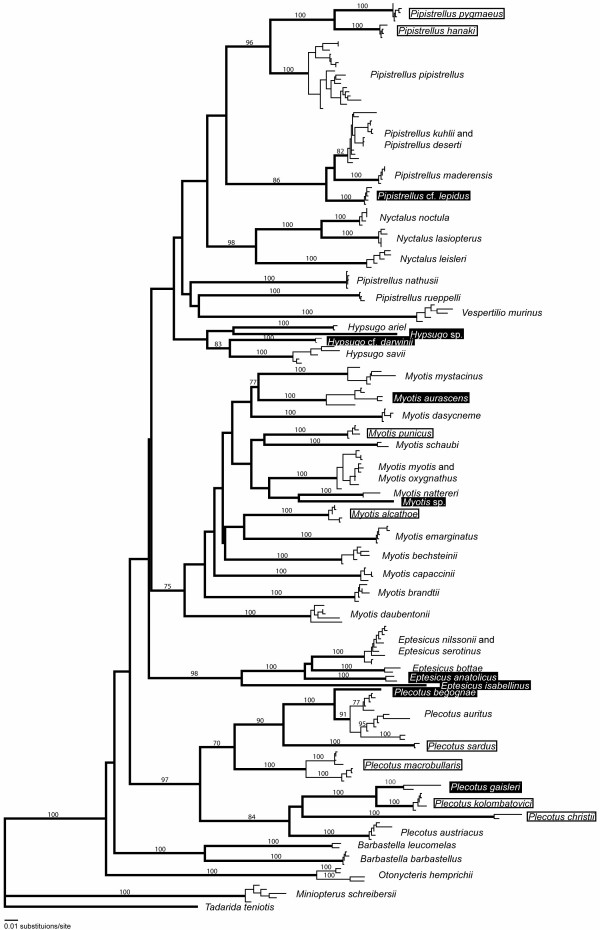
**Phylogeny of western Palaearctic vespertilionid bats**. Neighbor-joining (NJ) tree based on Kimura 2-parameter distances between mitochondrial *nd1 *sequences from 216 vespertilionid bats of the Western Palaearctic region. Thick branches lead to the different species while thin branches indicate intraspecific distances. Species names are encircled for species that were recently discovered with the help of DNA sequencing. Names of taxa for which a status as separate species is first proposed herein are highlighted by a black rectangle. *Tadarida teniotis *of the family Molossidae was used as an outgroup. Bootstrap support values of at least 70 are given above branches (5000 bootstrap replicates).

A recently compiled list of all mammal species of the world specifies a total of 44 vespertilionid bats species for the Western Palaearctic region [12]. One additional species was recently described (*Pipistrellus hanaki *[13]) and another was given species rank (*Plecotus christii *[14]). In this study we sequenced 900 base pairs of the mitochondrial *nd1 *gene [15] of 534 bats from 41 of the 46 described species [see Additional file 1]. This revealed species-specific DNA sequences that differed at least by 4% (uncorrected p distance) from sequences from other species in 816 of 820 pairwise species comparisons. Intraspecific variation was much lower than interspecific variation, which allowed a reliable genetic identification of most species (figure 1). Only four pairs of species showed genetic distances below 2.5% and species did not split in two monophyletic groups in all four pairs of species. Individuals of different species within these four species pairs occasionally shared the same sequence type. This lack of substantial sequence divergence between two species can have different reasons. Recent speciation or introgression of mitochondrial haplotypes are likely explanations for similar mitochondrial DNA sequences within the three species pairs *Eptesicus serotinus/E. nilssonii *[15], *Myotis myotis/M. oxygnathus *[16,17] and possibly also for *Pipistrellus kuhlii*/*P. deserti*. In contrast, the observed genetic similarity of only 0.3% sequence divergence and the similarity in morphology and echolocation calls between *Hypsugo ariel *and *H. bodenheimeri *is due to the fact that both taxa are likely to represent one single species as morphological differences are restricted to an additional cusp at the second upper incisor in *H. bodenheimeri *[18]. Therefore, we treated both taxa as a single species. In total, *nd1 *sequences of 40 investigated species allowed the unambiguous genetic identification of 34 species, which equals to 85%.

Substantial *nd1 *sequence divergence allowed also the distinction of several morphologically characterized taxa whose species status was questioned until recently [12,19]. Sequences of *Barbastella leucomelas *from the type locality on the Sinai Peninsula differed on average by 13.3% from sequences obtained from European *B. barbastellus*. In addition, morphologically characterized subspecies within four species (*Eptesicus bottae*, *E. serotinus*, *Plecotus auritus *and *P. kolombatovici *[see Additional file 2]) showed mean genetic distances between 5.8 and 15.2%. These examples illustrate that morphologically similar taxa can be genetically well differentiated and that the combination of morphological and mitochondrial DNA sequence datasets is a powerful approach to resolve taxonomic uncertainties. The congruence between slight morphological differences and substantial sequence divergence of at least 5% indicates distinct species. Nevertheless, only the sympatric occurrence of both taxa can finally proof the existence of true biological species [20].

Highly divergent mitochondrial lineages were found in four species. Most astounding were two bats in central Europe (southern Austria and northern Italy) that had been identified as *Myotis nattereri *according to external morphology. They differ in their sequences from other central European *M. nattereri *by on average 9.7%. Among 108 individuals of *Myotis mystacinus *from all over Europe we found two individuals in Bulgaria that carried another mtDNA haplotype that differed on average by 10.2% from typical *M. mystacinus*. Bats originally assigned to *Hypsugo savii *split even into three highly divergent lineages [see Additional file 2]. Finally, in *Pipistrellus kuhlii *a western (Europe, Morocco and Libya) and eastern (Israel, Syria and Iran) lineage can be distinguished, which differ on average by 5.2%.

Highly divergent mitochondrial lineages provide strong evidence for cryptic species diversity in western Palaearctic bats. Sequence differences of at least 5% clearly fall into the range of interspecific and even intergeneric differences in bats and other mammals [21]. They are concordant with genetic distances between recently discovered cryptic species pairs in bats [15,22-25]. A comparison of inter-specific genetic variation in the mitochondrial cytochrome *b *gene (*cob*) between sister taxa in four mammalian orders including bats led to the conclusion that sequence divergences of >5% are a powerful first indicator for unrecognized cryptic species [21]. The two mitochondrial protein coding genes *nd1 *and *cob *are known to evolve at a similar rate [16]. We thus applied a somehow arbitrary species delineation threshold of 5% *nd1 *sequence divergence, which suggests nine new bat species in the Western Palaearctic. This value drops to still six yet new species, if a more conservative threshold of 9% is applied. Both thresholds indicate at least three new species for Europe, one of them even occurring in central Europe. The validity of these newly proposed bat species will now be tested by analysing other characters like morphological, behavioural or ecological traits. However, the mitochondrial sequences constitute a reliable sorting criterion to search for species-specific phenotypic traits.

Although sequencing of the mitochondrial *nd1 *gene revealed evidence for substantial cryptic bat diversity in Europe, it is likely that further species exist that are morphologically and genetically difficult to recognize. First, mitochondrial lineages with less than 5% DNA sequence divergence as found within the species *Plecotus macrobullaris*, *Pipistrellus pipistrellus *and *Otonycteris hemprichii *might also represent distinct species [see Additional file 2]. Second, it is well known that DNA barcodes fail to distinguish recently diverged species [5]. A possible example are specimens of *Myotis mystacinus bulgaricus *from southeastern Europe (*Myotis aurascens sensu *Benda and Tsytsulina [26]) that differ from the nominate central European *M. mystacinus *in the chromosomal locations of nucleolus organizer regions [27] and in some morphological characters [26]. However, they show similar *nd1 *sequences that do not split into two separate lineages. Therefore, sequencing parts of the mitochondrial genome will always provide only the lower limit of true species diversity.

**Figure 2:**
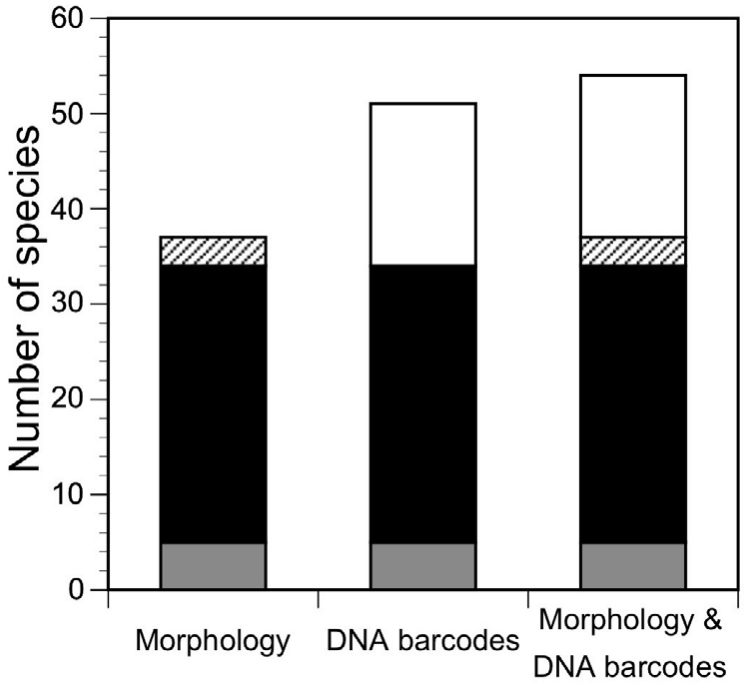
**Cryptic species diversity in western Palaearctic vespertilionid bats**. Number of vespertilionid bat species in the Western Palaearctic according to a morphological, a mtDNA sequencing or an integrative approach. Patterns distinguish genetically not investigated species (grey), morphologically differentiated species with distinct DNA sequences (black), species without a distinct DNA sequences (hatched) and morphologically cryptic species (white).

Prior to the emergence of DNA sequencing in taxonomy 37 morphological defined vespertilionid bat species were acknowledged for the Western Palaearctic realm. Now a total of 51 species can be distinguished according to mitochondrial *nd1 *sequences (figure 2). However, neither the morphological nor the genetic approach alone detected all species. It is the combination of both that results in a preliminary total of 54 species in the Western Palaearctic (figure 2). This clearly shows the need for an integrative taxonomy approach in which phenotypic and genetic data are combined [28]. Only external morphological characters will enable rapid species identification in the field, which is an essential requirement for behavioural, ecological and evolutionary studies or conservation programs (e.g. European Bat Agreement, EU Habitats Directive).

The raise in species number from 37 to 54 corresponds to an increase by almost 50%. New species were discovered in all major genera. Since Western Palaearctic bat species are among the best-studied taxonomic groups in terms of number of studied individuals, geographic coverage and applied techniques, we anticipate an enormous rise in bat species diversity in less studied areas like for example the tropics.

## Methods

### Sampling

Morphological measurements and a wing tissue sample with a diameter of 5 mm were taken from each individual before it was released. Tissue samples were immediately stored in 80% ethanol and kept at room temperature. The number of specimens and geographic coverage varied among taxa and ranged from one to 112 individuals [see Additional file 1]. To avoid a bias due to unequal sampling intensity, we kept only five sequences per major mitochondrial lineage. Sequences from different localities were given priority and sequences from one locality were chosen at random. This resulted in a total of 217 sequences that were included in genetic distance calculations.

### Genetic analysis

We sequenced 900 bp of the mitochondrial gene *nd1 *(NADH dehydrogenase subunit 1) because a large data set was already available and the *nd1 *gene in vespertilionid bats evolves at a similar evolutionary rate as the mitochondrial protein coding gene *cytb *[16]. Unfortunately, no data from bats were available for the mitochondrial gene for cytochrome oxidase, subunit 1 (*cox1*), which is commonly used in DNA barcoding. The protocols of DNA isolation, amplification and sequencing were previously published [15]. Sequence alignments were unequivocal. We found neither insertions, deletions nor stop codons that would have been expected for nuclear pseudogenes. Sequence differences of all divergent lineages were confirmed by sequencing 550 base pairs of a second mitochondrial gene, the gene for 16S rRNA, which was proposed as a standard DNA barcoding marker for vertebrates [29] [see Additional file 3]. Genetic distances (uncorrected p and Kimura 2-parameter with a gamma distribution shape parameter of 0.5) were calculated and illustrated in a neighbor-joining tree by using the computer program PAUP* 4.0b10 [30]. In the text we always refer to uncorrected p distances. All DNA sequences are available from GenBank: Accession numbers of previous studies [GenBank:AF065106, GenBank:AF401362, GenBank:AF401363–AF401366, GenBank:AF401369, GenBank:AF401372, GenBank:AF401373, GenBank:AF401375, GenBank:AF401376, GenBank:AF401378, GenBank:AF401382, GenBank:AF401390, GenBank:AF401393, GenBank:AF401395–AF401398, GenBank:AF401400, GenBank:AF401407, GenBank:AF401409–AF401411, GenBank:AF401414–AF401419, GenBank:AF401421, GenBank:AF401427, GenBank:AF401431–AF401436, GenBank:AF401439–AF401442, GenBank:AF401444, GenBank:AF401446, GenBank:AF401448, GenBank:AF401449, GenBank:AF401451, GenBank:AF401453–AF401457, GenBank:AF401461–AF401466, GenBank:AF401468–AF401477, GenBank:AF516269, GenBank:AF516270, GenBank:AF516272–AF516275, GenBank:AF516277, GenBank:AY027834–AY027837, GenBank:AY027840, GenBank:AY027846, GenBank:AY027848, GenBank:AY027851, GenBank:AY027853, GenBank:AY027856, GenBank:AY027858, GenBank:AY027859, GenBank:AY033950, GenBank:AY033955, GenBank:AY033984–AY033987, GenBank:AY699856, GenBank:AY699858, GenBank:AY699860] and of this study [GenBank:DQ914967–DQ915087].

## Competing interests

The author(s) declare that they have no competing interests.

## Supplementary Material

Additional file 1 Investigated species. A list of species, sample size and geographic origin of analysed bats from the Western Palaearctic realm. Click here for file

Additional file 2 Taxonomic comments. Taxonomic details are provided for newly proposed species and divergent mitochondrial lineages. Click here for file

Additional file 3 DNA sequence divergence. Mean pair-wise sequence divergences (uncorrected p distances) between Western Palaearctic vespertilionid bat species measured by sequencing the two mitochondrial genes *nd1 *and 16S rDNA. Sequence divergences between newly proposed cryptic species pairs are indicated. The two lowest genetic distances originate from the mean values of the species comparisons *Eptesicus serotinus/E. nilssonii *and *Myotis myotis/M. oxygnathus*. Click here for file
