# Supplementary material for: Molecular species identification boosts bat diversity
Source: Front Zool. 2007 Feb 12;4:4. doi: 10.1186/1742-9994-4-4 (PMC1802075; doi:10.1186/1742-9994-4-4)
Supplement: Additional file 1 — Investigated species. A list of species, sample size and geographic origin of analysed bats from the Western Palaearctic realm. [file 1742-9994-4-4-S1.rtf]

Table (electronic supplement) List of species, sample size and geographic origin of analyzed bats from the Western Palaearctic realm.

Species	Number of bats	Origin (number of bats)	
	sequenced	analyzed		

Outgroup	
Tadarida teniotis (Rafinesque, 1814)	1	1	Greece (1)	
				
Traditional species not investigated (lack of material)	
Myotis hajastanicus Argyropulo, 1939	0	0	-	
Myotis nipalensis Dobson, 1871	0	0	-	
Nyctalus azoreum (Thomas, 1901)	0	0	-	
Nycticeinops schlieffeni (Peters, 1859)	0	0	-	
Plecotus teneriffae Barrett-Hamilton, 1907	0	0	-	
				
Traditional species with characteristic mitochondrial DNA sequences	
Pipistrellus pipistrellus (Schreber, 1774)	62	17	Italy (3), Morocco (4), Spain (1), France (1), Germany (1), Greece (1), Turkey (4), Israel (2)	
Pipistrellus nathusii (Keyserling and Blasius, 1839)	10	5	Russia, Greece (2), Germany (2)	
Pipistrellus rueppellii (Fischer, 1829)	3	3	Morocco (3)	
Pipistrellus maderensis (Dobson, 1878)	7	5	Portugal (Madeira) (5)	
Nyctalus noctula (Schreber, 1774)	24	5	Germany (5)	
Nyctalus leisleri (Kuhl, 1817)	6	5	Greece (2), Turkey (1), Ireland (2)	
Nyctalus lasiopterus (Schreber, 1780)	5	5	Greece (1), Hungary (4)	
Hypsugo ariel (Thomas, 1904) (including H. bodenheimeri (Harrison, 1960))	4	2	Egypt (2)	
Hypsugo savii (Bonaparte, 1837)	9	5	Turkey (2), Croatia (1), Israel (1), Greece (1)	
Vespertilio murinus Linnaeus, 1758	5	5	Switzerland (1), Mongolia (1), Germany (2), Russia (1)	
Eptesicus bottae (Peters, 1869)	2	2	Egypt (1), Israel (1)	
Barbastella leucomelas (Cretzschmar, 1826)	2	2	Egypt (2)	
Barbastella barbastellus (Schreber, 1774)	4	4	Greece (1), Hungary (1), Germany (2)	
Myotis mystacinus (Kuhl, 1817)	112	5	Greece (1), Bulgaria (1), Israel (1), Germany (1), Morocco (1)	
Myotis brandtii (Eversmann, 1845)	31	5	Russia (1), Germany (1), Bulgaria (1), Hungary (1), Greece (1)	
Myotis emarginatus (Geoffroy, 1806)	7	5	Israel (2), Greece (1), Germany (1), Belgium (1)	
Myotis daubentonii (Kuhl, 1817)	12	5	Spain (1), Germany (2), Greece (1), Belarus (1)	
Myotis dasycneme (Boie, 1825)	4	4	Russia (1), Netherlands (1), Hungary (2)	
Myotis nattereri (Kuhl, 1817)	4	2	Greece (1), Hungary (1)	
Myotis schaubi Kormos, 1934	2	2	Iran (1), Turkey (1)	
Myotis capaccinii (Bonaparte, 1837)	4	4	Greece (3), Italy (1)	
Myotis bechsteinii (Kuhl, 1817)	5	5	Switzerland (1), Germany (3), Greece (1)	
Plecotus austriacus (Fischer, 1829)	12	5	Italy (1), Greece (2), Germany (1), Spain (1)	
Plecotus auritus (Linnaeus, 1758)	24	12	Switzerland (5), Ireland (1), Austria (1), Russia (1), Germany (1), Croatia (1), Italy (2)	
Otonycteris hemprichii Peters, 1859	4	4	Morocco (2), Israel (2)	
Miniopterus schreibersii (Kuhl, 1817)	6	5	Greece (1), Croatia (1), Serbia (1), Hungary (1), Spain (1)	
				
Traditional species pairs with similar DNA barcodes	
Pipistrellus kuhlii (Kuhl, 1817) plus 
Pipistrellus deserti Thomas, 1902*	
17	
13	
Greece (3), Italy (1), Morocco* (5), Libya* (4)	
Eptesicus serotinus (Schreber, 1774) plus	8	5	Hungary (1), Greece (2), Germany (2)	
Eptesicus nilssonii (Keyserling and Blasius, 1839)	5	5	Germany (3) Russia (1), Mongolia (1)	
Myotis myotis (Borkhausen, 1797) plus	8	5	Germany (1), Greece (1), Poland (3)	
Myotis oxygnathus Monticelli, 1885	5	5	Greece (4), Turkey (1)	
				
Morphologically cryptic bat species with characteristic mitochondrial DNA sequences	
Pipistrellus pygmaeus (Leach, 1825)	33	5	Greece (1), Sweden (1), Ukraine (1), Spain (1), Germany (1)	
Pipistrellus hanaki Hulva and Benda 2004	4	4	Libya (4)	
Myotis alcathoe von Helversen and Heller, 2001	19	5	Greece (3), Hungary (2)	
Myotis punicus Felten 1977	4	4	Morocco (3), Italy (1)	
Plecotus sardus Mucedda, Kiefer, Pidinchedda and Veith, 2002	2	2	Italy (2)	
Plecotus kolombatovici Dulic, 1980	13	5	Croatia (2), Greece (3)	
Plecotus macrobullaris (Kuzjakin, 1965)	14	8	Greece (4), Italy (1), Austria (2), Switzerland (2)	
Plecotus christii Gray, 1838	2	2	Egypt (1), Jordan (1)	
				
Newly proposed species with characteristic mitochondrial DNA sequences, which were traditionally regarded as subspecies according to morphology	
Eptesicus anatolicus Felten, 1971	2	2	Greece (2)	
Eptesicus isabellinus (Temminck, 1840)	1	1	Morocco (1)	
Plecotus begognae dePaz, 1994	1	1	Spain (1)	
Plecotus gaisleri Benda, Kiefer, Hanak and Veith, 2004	2	2	Libya (2)	
				
Newly proposed species (according to characteristic mitochondrial DNA sequences)	
Pipistrellus cf. lepidus Blyth, 1845	11	5	Turkey (1), Israel (1), Syria (1), Iran (2)	
Hypsugo sp.	1	1	Israel (1)	
Hypsugo cf. darwinii (Tomes, 1859)	2	2	Morocco (2)	
Myotis aurascens Kuzjakin, 1935	7	5	Bulgaria (2), unknown# (3)	
Myotis sp.	2	1	Austria (1)	
	534	217		

* identification of North-African bats were ambiguous; # sequences were retrieved from GenBank
