# Supplementary material for: Molecular species identification boosts bat diversity
Source: Front Zool. 2007 Feb 12;4:4. doi: 10.1186/1742-9994-4-4 (PMC1802075; doi:10.1186/1742-9994-4-4)
Supplement: Additional file 2 — Taxonomic comments. Taxonomic details are provided for newly proposed species and divergent mitochondrial lineages. [file 1742-9994-4-4-S2.rtf]

Taxonomic comments
Newly proposed species that were traditionally regarded as subspecies
Eptesicus isabellinus (Temminck, 1840): In Eptesicus serotinus the ND1 sequences of the traditional subspecies isabellinus [1] from NW-Africa and serotinus from Europe differed on average by 15.2%.

Eptesicus anatolicus Felten, 1971: Individuals initially assigned to Eptesicus bottae split into two mitochondrial lineages of 9.7% sequence divergence that correspond to the subspecies anatolicus from Asia Minor [2] and Greek islands and innesi from the Middle East. However, it cannot be ruled out that the latter does not represent the true E. bottae with the type locality in Yemen.

Plecotus gaisleri Benda, Kiefer, Hanak and Veith, 2004: North African and European representatives of P. kolombatovici showed sequence differences of 5.9%, suggesting that the North-African bats represent a valid species that should be named P. gaisleri [3].

Plecotus begognae dePaz, 1994: Within the genetically diverse Plecotus auritus the subspecies begognae from the Iberian Peninsula [4] differs from other European individuals by 5.8%.

Newly proposed species
Pipistrellus cf. lepidus Blyth, 1845: Individuals of Pipistrellus kuhlii that were collected in Israel, Syria and Iran differ on average by 5.2% from western representatives of Pipistrellus kuhlii. According to the geographic origin and morphological characters the eastern animals are likely representatives of Pipistrellus lepidus Blyth, 1845. This taxon was regarded as a synonym of P. kuhlii in the past [5-8].

Hypsugo cf. darwinii Tomes, 1859: A bat caught in Morocco differed from European H. savii by at least 9.6%. Similar sequence differences were observed between animals from the Iberian Peninsula and from the Canary Islands [7]. We provisionally use the name Hypsugo cf. darwinii (Tomes, 1859) because this taxon was described from specimen collected on the Canary Islands.

Hypsugo sp.: The mitochondrial DNA sequence from one "Hypsugo savii" from Israel differed by 13.8% from other sequences of this species from the same area.

Myotis sp.: Two bats from Austria (Carinthia) and Northern Italy that were identified as Myotis nattereri according to external morphological characters, but differed in their sequences from other central European M. nattereri by on average 9.7% (min. 8.7%). So far morphological differences between both mitochondrial lineages could not be investigated, because both bats with the highly divergent ND1 sequence were immediately released after sampling and hence prior to DNA sequencing.

Myotis aurascens Kuzjakin, 1935: Among 114 individuals of Myotis mystacinus from all over Europe we caught two individuals in Bulgaria that carried another mtDNA haplotype, which differed by 10.2% from M. mystacinus haplotypes, including the subspecies bulgaricus from the Balkans. This lineage shows similar haplotypes to "Myotis aurascens" from the Caucasus and Asia published in GenBank (Accession numbers AY699856, AY699858 and AY699860). The situation on the Balkans is quite complicated because bats of the morphotype Myotis aurascens sensu Benda and Tsytsulina [9] from Bulgaria and Greece were genetically identified as M. mystacinus bulgaricus, while our genetically established true Myotis aurascens show the same ND1-sequence as bats from the Caucasus area also included in Myotis aurascens sensu Benda and Tsytsulina [9]. We conclude from this that Benda and Tsytsulina [9] erroneously lumped both, Myotis aurascens and Myotis mystacinus bulgaricus into one species.

Additional divergent mitochondrial lineages
Plecotus macrobullaris (Kuzjakin, 1965): ND1 sequences from bats caught in the Alps and in Greece differed on average by 4.6% (range 4.3 - 4.8%). This supports the distinction of the two subspecies macrobullaris (Balkan) and alpinus (Central Europe) and raises the question, whether both taxa interbreed in contact zones that might exist in the northern Balkan or in the eastern parts of the Alps.

Pipistrellus pipistrellus (Schreber, 1774): Three distinct ND1 lineages were found that differed in up to 4.5%: the wide spread subspecies pipistrellus, the subspecies aladdin (bats from eastern Turkey and Israel) [10, 11] and a third lineage from Morocco [12] and Sardinia.

Otonycteris hemprichii Peters, 1859: ND1 sequences of bats from Morocco differed by at least 3.3% from sequences obtained from individuals caught in Israel.

Plecotus auritus (Linnaeus, 1758): Three distinct ND1 lineages were found that formed geographic clusters (a western and eastern lineage and one lineage on Sardinia). Pairwise sequence comparisons revealed up to 5.2% sequence divergence but mean sequence differences between the three clusters were below 5%.

References
1.	Harrison DL: Observations on the north African serotine bat, Eptesicus serotinus isabellinus (Mammalia: Chiroptera). Zoologische Mededelingen, Rijksmuseum van Natuurlijke Historie te Leiden 1963, 38:207-212.
2.	Felten H: Eine neue Art der Fledermaus-Gattung Eptesicus aus Kleinasien. Senckenbergiana biologica 1971, 52:371-376.
3.	Benda P, Kiefer A, Hanak V, Veith M: Systematic status of African populations of long-eared bats, genus Plecotus (Mammalia: Chrioptera). Folia Zoologica 2004, 53:1-47.
4.	de Paz O: Systematic position of Plecotus from the Iberian Peninsula. Mammalia 1994, 58:423-432.
5.	Koopman KF: Chiroptera: Systematics. Handbook of Zoology 1994, 8:1-217.
6.	Simmons NB: Order Chiroptera. In Mammal species of the World: a taxonomic and geographic reference. Edited by Wilson DE, Reeder DM. Baltimore, MD: Johns Hopkins University Press; 2005: 312-529
7.	Pestano J, Brown RP, Suarez NM, Fajardo S: Phylogeography of pipistrelle-like bats within the Canary Islands, based on mtDNA sequences. Mol Phylogenet Evol 2003, 26:56-63.
8.	Horáček I, Hanak V, Gaisler J: Bats of the Palaearctic region: A taxonomic and biogeographic review. In Proceedings of the VIIIth European bat research symposium. Volume Vol. I Approaches to biogeography and ecology of bats. Edited by Woloszyn BW. Krakow, Poland: Institute of Systematics and Evolution of Animals; 2000: 11-157
9.	Benda P, Tsytsulina KA: Taxonomic revision of Myotis mystacinus group (Mammalia: Chiroptera) in the western Palearctic. Acta Soc Zool Bohem 2000, 64:331-398.
10.	Neuhauser HN, DeBlase AF: The status of Pipistrellus aladdin from Central Asia. Mammalia 1971, 35:273-282.
11.	Albayrak I: A new record of Pipistrellus pipistrellus aladdin for Turkey. Commun Fac Sci Univ Ank Series C 1987, 5:31-37.
12.	Benda P, Hulva P, Gaisler J: Systematic status of African populations of Pipistrellus pipistrellus complex (Chiroptera: Vespertilionidae), with a description of a new species from Cyrenaica, Libya. Acta Chiropterologica 2004, 6:193-217.
