# Supplementary figures and images for: Molecular species identification boosts bat diversity
Source: Front Zool. 2007 Feb 12;4:4. doi: 10.1186/1742-9994-4-4 (PMC1802075; doi:10.1186/1742-9994-4-4)

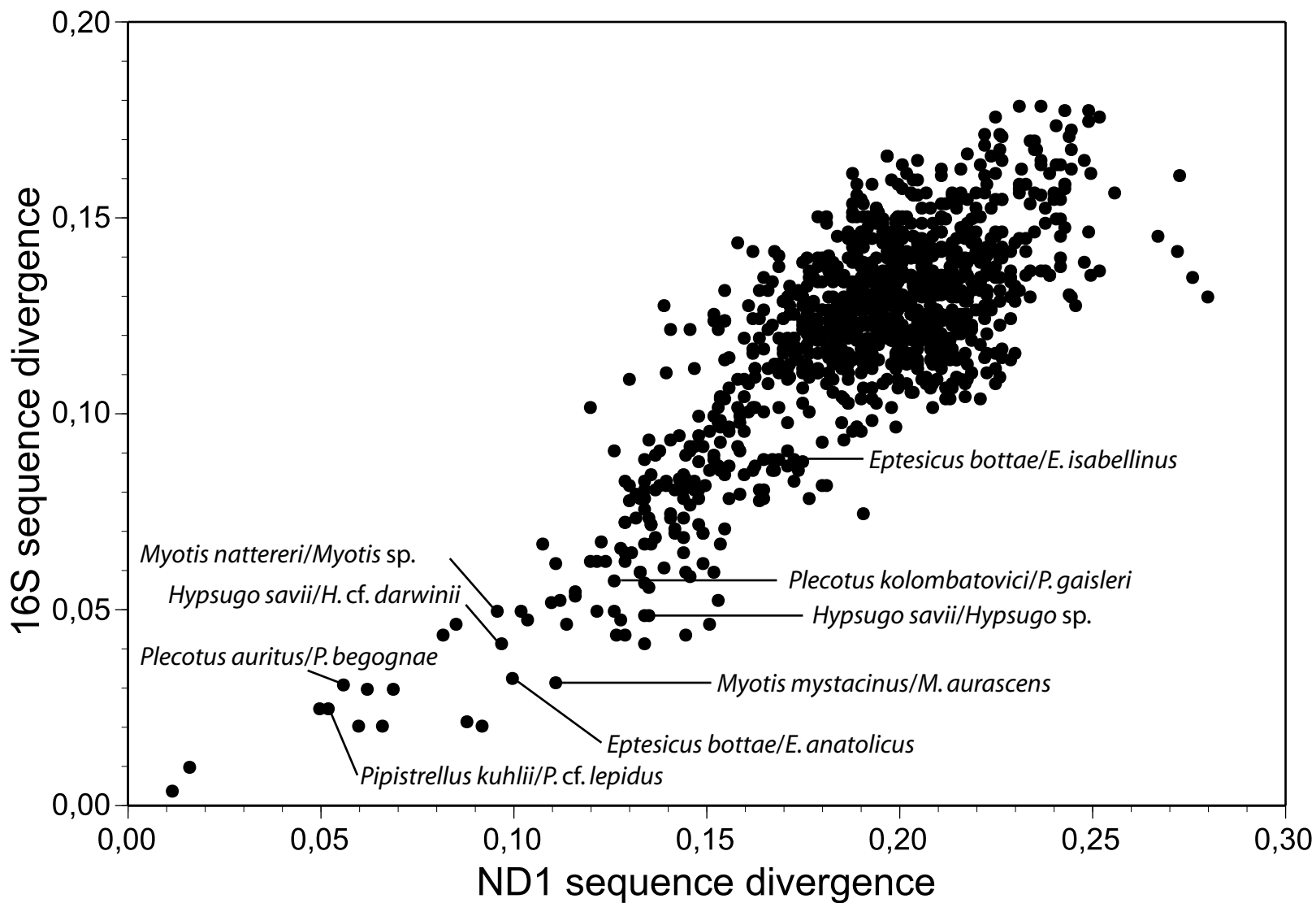

Supplement: Additional file 3 — DNA sequence divergence. Mean pair-wise sequence divergences (uncorrected p distances) between Western Palaearctic vespertilionid bat species measured by sequencing the two mitochondrial genes nd1 and 16S rDNA. Sequence divergences between newly proposed cryptic species pairs are indicated. The two lowest genetic distances originate from the mean values of the species comparisons Eptesicus serotinus/E. nilssonii and Myotis myotis/M. oxygnathus. [file 1742-9994-4-4-S3.pdf]
